# A Tempered Rationalism for a Tempered Yuck Factor—Using Disgust in Bioethics

**DOI:** 10.1007/s41649-023-00278-x

**Published:** 2024-03-28

**Authors:** Konstantin Eckl, Konstantin Deininger

**Affiliations:** 1 Unit of Ethics and Human-Animal Studies, Messerli Research Institute, University of Veterinary Medicine Vienna, Medical University of Vienna, University of Vienna, Vienna, Austria; 2 Department of Philosophy, Vienna Doctoral School of Philosophy, University of Vienna, Vienna, Austria

**Keywords:** Rationalism, Disgust, Yuck factor, Mary Midgley, Animal ethics, Farm animal breeding

## Abstract

When it comes to invasive manipulation of animals on the biological level, reactions of disgust are common and often influential on people’s moral judgments. As a case in point, the Belgian Blue, a breed of hyper-enhanced cattle which will serve as a case study for the present article, has historically been met with revulsion. Traditionally, in bio- and animal ethics, this ‘yuck factor,’ has been denied any productive role in proper moral justification, since rationalism is still a dominant paradigm in those disciplines. This is not surprising since rationalism offers the fulfilment of certain expectations we have of morality, like universality, intersubjective communicability, and objectivity. Increasingly, however, the preconceptions of rationalism have been brought into question, both through empirical as well as philosophical insights. In this paper, we will explore a way in which researchers who are, accordingly, critical of rationalism, and who wish to take seriously the role disgust plays in the formation of moral judgments when it comes to biological manipulation of animals, can do so without abandoning those virtues of rationalism which make it such an appealing position. We will do so by offering what we call a ‘tempered’ kind of rationalism, that is, one which conceives of rationality in the terms of Mary Midgley, not as distinct from, but as a possible function of, well-ordered emotion.

## Introduction

In this paper, we defend the possibility of appeals to disgust playing a productive role in moral justification, particularly as it intersects with animal ethics. The view we advance is designed to account for criticisms of rationalist dismissals of emotion while at the same time retaining rationalism’s virtues, i.e., intersubjective communicability and objectivity.

We claim that while bioethicists have good reason to question the assumption of rationalism commonly at play in the treatment of moral emotions in general and disgust^1^ in particular, the wholesale rejection of rationalism leads to undesirable consequences for the possibility of making ethical recommendations as such. Instead, we advocate for a tempered version of rationalism which retains rationalism’s demands on morality but revises its psychological picture.

In arguing for this position, we choose to focus on the emotion of disgust. The reason for this is, firstly, that the topic for which we chiefly want to provide guidance and which will serve as the main case study of the text is the issue of invasive interference with animal bodies on the biological level. Such interference encompasses, e.g., genetic engineering, directed livestock breeding, and cloning. In these cases, reactions of disgust are both common (Olofsson and Öhman 2016; Blancke et al. 2015) and often influential on people’s moral judgments (Rozin et al. 2008; for a dissenting opinion, cf. Sanyal et al. 2023).

Secondly, disgust and similar ‘gut level,’ ‘hair trigger’ emotions face stronger, and qualitatively different opposition than do more obviously cognitively loaded and oft-evoked emotions like empathy. The latter are defined with clear failure conditions and objectively discernible conditions of appropriateness which disgust largely lacks. An instance of feeling empathy, for example, can unproblematically be said to go wrong if, e.g., it fails entirely to represent the mental states of its target (cf. Gruen 2015, 83–89). This is not the case with emotions like disgust and repugnance, which seem to exhibit no obvious failure conditions, i.e., conditions where the emotion can unproblematically said to be misfiring. As such, the challenge posed by disgust’s seeming incompatibility with rationalism is one in need of special elucidation.

The third reason for our focus on disgust is that within bioethics, there is already a discussion on what is commonly referred to as the ‘yuck factor.’ As we use it, the yuck factor, or ‘wisdom of repugnance’ denotes the influence of disgust or repugnance on moral judgments insofar it is productively involved in, rather than merely distorting, inquiry into the proper justification of moral judgments. Within this discussion, we identify two problematic extreme positions—rationalism, which rejects the yuck factor outright, on the one hand, and non- or even a-rationalist positions which give disgust normative and sometimes even epistemic powers unassailable to rationality (e.g., Kass 1997).

The guiding example we will use for a case of invasive interference with animal bodies will be that of the Belgian Blue, a breed of cow which is an extremely ‘enhanced’ type of cattle which diverges dramatically from our picture of the ‘normal’ cow. We choose the Belgian Blue, and visible breeding in general, as illustration for the yuck factor, as it is the more tangible case. Our conclusions apply also to less visible cases like the genetic engineering of hornless cattle and the more conceptual disgust responses associated with them.

We argue that the ‘yuck’-responses which are common towards the Belgian Blue (Olofsson and Öhman 2016) and other such cases should neither be rejected outright nor uncritically accepted as grounding moral justification. Rather, we argue with Mary Midgley (2003) that they should be taken seriously as part of a self-evaluating cognitive-emotional apparatus which is rational just then when it coheres well with reality. A response of the form of “too many animal manipulations […] it is sick!” (Olofsson and Öhman 2016, 193) or even just of ‘yuck!’ is intelligible and criticisable for its fittingness to reality or what Klaus Scherer (2011) calls “the realism of the underlying appraisal.” When rationalists dismiss emotions, this, we claim, is what they are after: they want to keep morality the purview of a reality-facing capacity capable of getting us to intersubjectively communicable and subject-invariant correct judgments. They are right, we think, in wanting this, but wrong about where to find it—there is little hope to get to our best possible picture of reality without heeding the more affective aspects of our cognition, and heeding them as part, rather than as mere objects of, our deliberative process. In short, we shall claim that the problem with rationalism is not its demands on morality but its anthropology.

## Disgust and Interference with Animal Bodies

The Belgian Blue is a breed of cows whose continued existence is dependent on human intervention. Belgian Blues are ‘double muscled,’ meaning they produce much more muscles than would be usual for ordinary cattle. This causes them to have a higher agricultural yield, but also means that a number of capacities usually found in cows are limited for them. For example, they have a hard time birthing naturally, and calves are instead cut from the mother by means of C-section in over 90% of cases (Vandenheede et al. 2001). They are also less able to move on their legs and occasionally are born with enlarged tongues which make it hard for the calf to suckle on its mother’s teats (Sartelet et al. 2014).

**Fig. 1:**
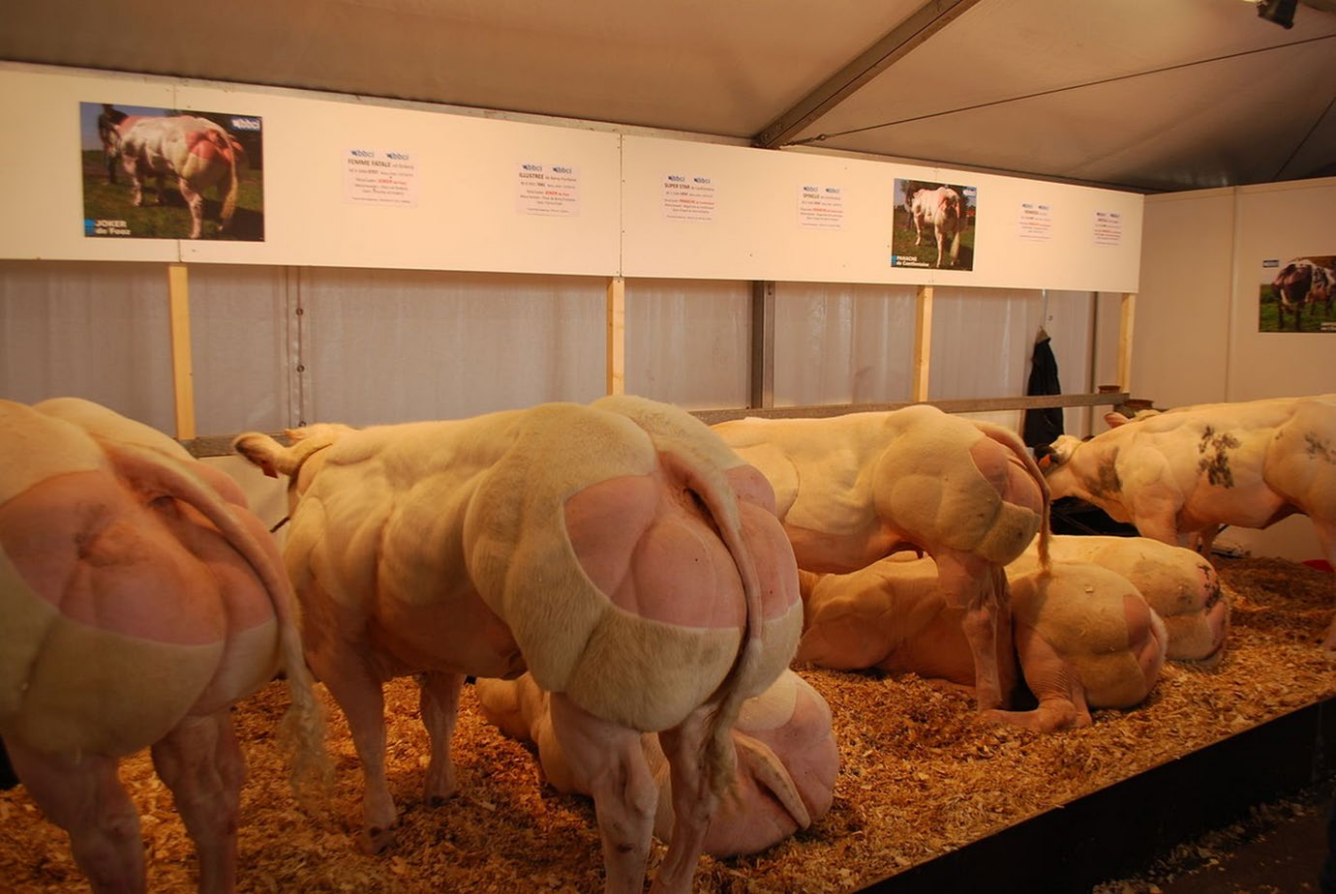
The distinctive rump of the Belgian Blue (Housen 2011)

Seeing a double-muscled Belgian Blue in the flesh will immediately show the difference between them and other breeds of cows. Their skin is tightly packed with muscles so that one can clearly see each individual muscle move. Often, thick veins can be seen under the skin, and what is described as “large, bulky muscles, especially of the shoulder and rump” (Valentine 2017; Fig. 1) give it a distinct profile compared to non-double-muscled breeds.

In this description, the reader may have found some things they are disgusted by: Perhaps imagining the sight of the animal packed with superfluous muscle mass causes revulsion. Maybe one is bothered by the thought of the calves having to be cut from their mothers, or by the idea of reproducing what is ostensibly a disability to increase agricultural yield.

Some of these feelings may even have coincided in the reader with a moral judgment—thoughts of the form ‘this is disgusting—this is not okay.’ In some of these cases, it will seem highly inappropriate to use the reaction of revulsion to ground a moral judgment. The fact that an animal is veiny in an unappetizing way, for example, should probably not make us condemn its breeding as unethical. In other cases, such as the deliberate proliferation of a reproductive disability, however, it is not so clear. The revulsion we feel at the thought of calves being cut from their mothers, for example, seems to raise legitimate ethical concerns.

In these cases, our affective response coinciding with moral judgment does not alone give us conclusive reason to assume that this emotion is appropriate to be used in the justification of our ethical evaluations. It nevertheless urges us to consider the possibility and therefore justifies us to ask: Can we afford disgust a productive role in moral justification?

## The Rationalist Response to Disgust

In bioethics, especially when it comes to human interference with animals on a biological level, the question of whether feelings of disgust can be an appropriate justification for moral judgments can have wide-reaching implications. If our revulsion at the sight of the Belgian Blue should be treated as counting against its proliferation in moral argument, then our emotional responses have the potential to have societal or even legal consequences. As an example, Leon Kass played a significant role in the discussion on the banning of human cloning, relying on an appeal to disgust in his influential (1997) paper “The Wisdom of Repugnance: Why we Should Ban the Cloning of Humans.”

For the purposes of this paper, we will refer to positions like that of Kass, which give disgust (or any emotion or feeling) a justifying role in moral judgments that is unassailable by rationality as the ‘non-rationalist’ stance. This will be contrasted with the ‘rationalists’ who argue that ethical decisions can only be justified if they agree with precepts of reason. These rationalist philosophers hold that “[o]ur emotions often motivate us to do what is right, but they are equally likely to motivate us to do what is wrong. In making ethical decisions, it is our ability to reason—not our ability to feel […] that should play a crucial role” (Singer 2016). Although we can adjust our character and emotional responses to align with ethical conduct, determining the nature of such conduct, as per this perspective, falls entirely within the domain of rationality, which is conceived as a psychological capacity wholly separate from and independent of emotions. Although different rationalists have different conceptions of rationality, and rarely make them explicit, what they have in common is that they treat rationality as a tool for approximating objectivity. As we use the word in this text, for something to be objective, it is enough for it to be intersubjectively communicable and theoretically intelligible by any competent inquirer. This is what, to rationalists, rationality does: it gives us access to structures in the world which can be discovered in the same way by anyone endowed with that capacity.^2^ Examples of such structures given by rationalists are things like mathematical rules (Lazari-Radek and Singer 2016, 182) or logical laws (Parfit 2013, xxv).

Rationalism, in the sense we use it, can then be defined as consisting of two theses, a psychological one and one about the nature of moral justification:

[PT]: Humans have a capacity which is wholly separate from and independent of emotions, and which has the function of accessing objective structures in the world. This capacity is rationality.

and

[MT]: Whatever capacity which gives us such unclouded access to the objective structure of the world, is the only reliable epistemic resource for getting to correct and justified moral beliefs.

The construal we have just given is likely to exclude some positions which ordinarily may be included under that label. Since we are, in this article, arguing for a “tempered” version of rationalism, we are more than happy to accept that there are positions which are able to moderate the strictness of these rationalist theses—one of these we will be talking about ourselves in the form of Mary Midgley further down.^3^ Our, stricter, sense of rationalism is far from a caricature, however. For Peter Singer and Katarzyna de Lazari-Radek, for example, emotion is at most a “concomitant or expression” of the rational moral judgment which necessarily “comes first” (Lazari-Radek and Singer 2016, 65). In *Animal Liberation*, Singer plainly states: “The ultimate justification for opposition [to animal cruelty] is not emotional” (Singer 1975, xxii). Similarly, Tom Regan rejects a role for emotion in moral theories outright, asking: “How […] could one conceivably offer a theory of animal rights based on appeals to emotion? What could such a ‘theory’ possibly maintain?” (Regan 2001, 63), and rejecting care ethics partially because of a “lack of a rule of reason to regulate compassion” (Regan 2004, 80–81).

Rationalism has been influential in moral philosophy at least in part due to its ability to provide us with a conception of morality which fulfils some deeply held intuitions. Firstly, by appealing to the shared capacity of rationality, it allows for intersubjective communication of moral judgments. If moral truths can be accessed rationally, and if all moral agents are rational, then the hope is that everyone can arrive at a correct understanding of what is morally right, irrespective of subjective feelings or particular values. Additionally, since rationality is here conceived as being concerned with objective properties and structures of a common world, the rationalist approach to morality allows for moral disagreements to be conceived as genuine disagreements with the possibility of one party being wrong and the other being right, and therefore vindicated in their beliefs. This avoids the worry associated with some sentimentalist approaches to morality, where disagreements cannot be resolved if both parties have the appropriate feelings or attitudes. By the same token, rationalism thus conceived assures that morality is non-arbitrary, as it is concerned with structures objectively instantiated in, or inherent to, a shared world and governed by logical principles held in common by all relevant agents.

Noell Birondo sums up the intuitions at the root of rationalist thinking in morality as “the deep-seated conviction that the requirement of morality are both *universal* (applying equally to everyone) and *necessary* (such that moral requirements could not have been different than they actually are).” (Birondo 2017, 2) This conviction is what “motivated early rationalists to [assert] that the provenance of moral requirements lies […] in reason” (ibid.)

Rationalism’s ability to satisfy those intuitions is especially relevant for bioethics and animal ethics, because it enables us to straightforwardly make sense of what Henry Rydenfelt calls”controversial views.” Controversial views are views which “[go] against the views of the majority and are typically voiced […] when there is a common or widely shared position that the controversial view aspires to contest” (Rydenfelt 2023).

What makes discourses around controversial views special is that in them, ethical minority positions which are often met with strong negative emotional intuitions, are taken to be *prima facie* possibly correct and worthy of discussion. This points to the fact that the assumption at play in such discourse is that the question about the correctness of a moral judgment is not dependent on such emotional intuitions, but subject to intersubjectively communicable discussion with the possibility of a definite correct or incorrect answer. Rydenfelt takes these assumptions to point to realist commitments in such discourses, though we are content with pointing out that they can be cashed in well by rationalism.

As Rydenfelt points out, controversial views are a central topic of bioethics. As it intersects with animal ethics, this centrality is likely to be even more pronounced. In animal ethics, it is often the case that moral judgments are defended which go against the prevailing intuitions of a discipline or a society. Animal ethicists frequently aim at such controversial recommendations as radical veganism (Huemer 2019), the protection of wild animals from predators (McMahan 2010), or even the total equality of humans and animals (Francione 2008). In order to defend such positions which will often invoke negative emotional responses in non-philosophers, it is certainly advantageous to argue from a vantage point independent of those emotional intuitions, and with a kind of objectivity which does not depend on general agreement. Accordingly, in animal ethics, rationalism still plays a prominent role, especially in the two most prominent traditions within it, utilitarian welfare ethics (Singer 2011) and animal rights ethics (Regan 1983).

Under this popular approach, the role disgust plays in moral justification is clear: It plays none at all. Any feelings of disgust towards the Belgian Blue are entirely immaterial to our moral evaluation of it and its breeding. Such affective responses are here seen as artifacts of evolutionary adaptations which are useful for orienting ourselves in an environment as organisms, but which may or may not point us in the direction of correct moral judgments. Those judgments themselves, if they are to be reliable, ought to be arrived at through dispassionate reasoning, the more dispassionate the more reliable. The rationalist response is, then, simple, universalizable, and popular. But despite its popularity, this kind of rationalism is not without problems.

### Rationalism Under Fire

As compelling as the rationalist approach may appear, it has recently increasingly come under fire from multiple directions. Criticism has come both from an empirical as well as from a more philosophical side. From the empirical side, the chief criticisms to rationalism have come from psychology and neuroscience. Many empirically minded philosophers (Haidt 2001; Prinz 2009; Churchland 2008; see for a somewhat dissenting opinion Greene 2013) point out that in moral deliberation rationality seems to play a secondary role. They point to experiments in which changes to a situation or environment which we do not consciously notice, and which we would not give moral weight if we did, have a profound impact on our moral decision-making, and to neuro-imagining which shows that in moral decisions, areas associated with emotion or ‘hot’ cognition are activated when making moral decisions. This, they claim, calls into doubt the rationalists’ idea of morality as a practice chiefly subject to rational examination. Instead, moral judgments are made by use of emotions and in large parts pre-consciously.

Such empirical evidence, rationalists can retort, is not necessarily an argument against the rationalists’ claims about morality and its justification. They are claims about the actual, observed, practice of moral decision-making, but not about the correct or proper results of such practice. Rationalists may concede that when deciding whether, e.g., the breeding of Belgian Blues is permissible, we are often guided by our disgust; but this, they could contend, is no reason to claim that the decision’s being guided by disgust does not disqualify it from being well-justified. They could still claim that even though it is not usually done, the correct way of finding out whether it is alright to deliberately perpetuate the existence of double-muscled cows is by use of the capacity of rationality, and not of emotion.

Even so, we may say that the apparent fact that such purely rational moral deliberation, if it is possible at all, seems to be rare, is already reason to doubt how plausible such an approach is. Even more damning, however, is the second main objection from the empirical side of things, which regards the existence of exactly that capacity which lies at the centre of rationalism. In contemporary cognitive science, it is often questioned whether we can really speak of a unified, distinct capacity of ‘rationality’ which is wholly independent of emotions and which can be used to give us somehow more accurate and less biased information about the world than the more affective aspects of our cognition. Increasingly, integrative views of rationality and emotion are being advanced, where rational thought relies on emotions to properly work, or where rationality is understood as a possible function of well-functioning emotions (e.g., Scherer 2011; Garcés and Finkel 2019). Even the conception of rationality on which most of the neuroethicists rely, the two-track-theory of mind, which affords emotions a large part in human cognition but still leaves open an independent realm of rational cognition, is increasingly put into doubt as overly simplistic in favour of conceptions with much less well-defined distinctions between the ‘two’ capacities (Melnikoff and Bargh 2018; Mercier and Sperber 2017).

The very real possibility that the capacity at the centre of the rationalist approach may not even exist in the way they conceive it is a further strong point for questioning the plausibility of the rationalist approach to moral theory. Related to these empirical points, there are also philosophical challenges to rationalism, including from the side of animal ethicists. This is particularly important as we are interested in the phenomenon of the yuck factor as it pertains to animal bodies as targets of human interventions. As noted above, rationalistic accounts of philosophers like Singer and Regan are still predominant (for more recent rationalist accounts see, e.g., Kagan 2019; McMahan 2002). Nevertheless, the strictly rationalistic account has been challenged early on for neglecting emotions and their moral value (e.g. Diamond 1978; Donovan 1996; Midgley 1983). Critics claim that such a rationalistic account distorts how our moral lives actually are—we are not convinced of how we ought to act by ‘our brain’ but also motivated by ‘our heart’ (*pace* Diamond 1995, chap. 11). At the core of such a critique lies that reason is not a single, purified faculty in man but that reason and emotions inform each other (e.g., Gruen 2015; Aaltola 2018; Luke 1992).

## Two Ways of Accounting for ‘the Yuck Factor’

For better or worse then, it appears no longer plausible to simply dismiss the role of emotion in moral justification out of hand simply by asserting the truth of rationalism. While rationalism as a position is far from refuted, there are certainly more than enough grounds to consider its alternatives.

### The Yuck Factor

For disciplines such as bioethics and animal ethics, which we have said above often rely on many of the assumptions which rationalism seeks to vindicate, moving away from that position comes with the danger of having to give up those assumptions. Take for example Leon R. Kass whom we have already mentioned as an example of someone who would afford disgust a justificatory role in moral deliberation. His aforementioned 1997 paper on the wisdom of repugnance represents the genesis of what nowadays is often called ‘the yuck factor’—the idea that disgust, or repugnance, or a similar pre-reflective feeling or emotion of aversion not only plays a role in moral deliberation but does so in a way that can give us productive insights into what should or should not be considered morally acceptable.

Kass uses this idea in response to the cloning of the sheep Dolly a year prior to the publication of his article, and with the intention of advocating for strict bans on the cloning of humans. Kass appeals to disgust as morally relevant evidence:

To pollution and perversion, the fitting response can only be horror and revulsion; and conversely, *generalized horror and revulsion are prima facie evidence of foulness and violation*. (Kass 1997, 689, emphasis ours)

Note that this, the taking of emotional responses such as disgust as *evidence* for the existence of moral violation is not prima facie incompatible with rationalism as we have defined it. A rationalist could argue that the results of rational deliberation often align with our emotional and affective responses because morality aims to maximize social cohesion, and we are evolutionarily adapted to negatively respond to factors that hinder such cohesion. In this view, emotional reactions can provide evidence that we are on the right track, but the ultimate determination of correctness is still based on rationality alone. The justificatory work is still done entirely rationally. Merely taking these affective responses as evidence, which then will still have to be rationally examined for whether what they point to is morally justified, is then not in conflict with rationalism. Kass, however, goes further than that:

[R]epugnance need not stand naked before the bar of reason. The wisdom of our horror at human cloning can be partially articulated, even if this is finally one of those instances about which the heart has its reasons *that reason cannot entirely know*. (Kass 1997, 689; emphasis ours)

Here, Kass parts with rationalism. He denies that all moral justification needs to, in the last instance, be rationally justifiable. To Kass, there is “deep wisdom beyond reason’s power to fully articulate” (1997, 687) which can only be accessed through the correct kind of repugnance. Kass conceives of rationality as largely a tool for problem-solving which not only does not give us access to objective reality, but often “[purchases] intelligibility and clarity […] at the cost of abstraction and distortion” (Kass 1990, 7). He, then, neither subscribes to the image of rationality which rationalism assumes, nor does he credit it as the sole source of moral justification. He rejects both theses of rationalism as we have defined it.

Note that, although Kass is not a rationalist, in the quotations we have given he still treats morality in the way that seems to be implied by, e.g., the discourse around controversial views. That means that for Kass, it is a subject-invariant fact—a piece of ‘deep wisdom’—that cloning is wrong. Our disgust towards it is sufficient evidence to that effect, which to a degree can be bolstered by rational argument but does not need to be fully rationally justifiable.

What he does not seem to be saying is that whenever we are disgusted, we are right to condemn something morally, or that, just because someone does not share Kass’ own reprehension towards cloning, that would exempt them from the moral obligation to refrain from cloning humans. Their lack of revulsion would not be evidence for Kass’ being wrong about his moral assessment but rather for that person not reacting properly to a moral outrage (perhaps due to a failure in education [compare Kass 1990, 9]). As such, under this view, one can take one’s disgust of the Belgian Blue to ground one’s moral judgment and try to convince others by persuasively trying to affect in them that same disgust; but if others do not react with the correct kind of revulsion even after such persuasion, then, presumably, it is their loss: They are unable to access the wisdom revealed by our disgust.

### Disgust Revealing versus Constituting Moral Facts

This alone will give some of us pause—the intersubjective communicability of the justification of our moral judgments seems essential for a productive ethics of any kind. Without the ability to communicate why a moral judgment is correct or not, ethical recommendations are unlikely to find purchase amongst an ethicist’s target audience. But even if we are ready to give up the ability to reach people with the wrong affective profile, the question remains: why should we trust our own emotional responses over those of others? The problems of this strong version of the yuck factor is summed up by Devolder et al. (2021). They criticise the use of “people’s intuitive disdain for human-animal chimeras as evidence that there is something wrong with creating them, *even if we cannot articulate what the problem is*” (2021, 436) by pointing out that:

First, different people have different intuitive [affective] moral responses to the same issue. Secondly, if we settle for accepting our own moral intuitions, regardless of whether they can be given any rational basis, then we might find ourselves with no basis for rejecting the moral intuitions of those who condone, for example, racism and slavery. (ibid.)

In other words, taking disgust as our ultimate guide in ethical evaluation presents us with a dilemma: We are left with either a) competing claims to moral correctness which are incommunicable and practically irresolvable or with b) a situation where all moral claims are equally valid as long as they arise from the relevant moral emotions. Faced with this dilemma, Kass has picked option a), in line with the presupposition of a bioethics with the aim of making definite recommendations for and against certain practices. But there remains a second horn to the dilemma which is hard to ignore. For, while Kass sticks to the idea of disgust *revealing* to us some fundamental insights into the moral states of affairs not otherwise accessible to our faculty of rationality, another interpretation of a strong yuck factor is that of such disgust (or other emotional responses) *constituting* those moral states of affairs. This is the position of emotionalists such as Jesse J. Prinz (2009), and it is a position with some distinct advantages over that of Kass. Its main advantage is that it makes do without attributing to the emotion of disgust an epistemic power which is not rationally assailable and which may therefore appear downright mystical if we take seriously its appeals to “reasons which reason cannot entirely know” (Kass 1997, 689).

If our response to disgust is indeed good evidence for our moral convictions being justified, and if that evidence cannot, in the last instance, be rationally refuted, then it would be reasonable to hold that our disgust-response is not only evidence, but *grounding* for justified moral belief. Under this interpretation, rationality’s inability to know the “reasons of the heart” is because there are no further justifying facts to *be* known. All that is needed for a moral judgment to be justified is its arising from the right moral feeling. This would be the more parsimonious way of interpreting an appropriate justificatory role of disgust in moral deliberation—rather than assuming some otherwise opaque moral matters of fact our affective responses reveal to us, those affective responses *are* the moral matters of fact.

This would entail embracing “arationalism, subjectivism, and relativism” (Prinz 2009, 2). With this, Prinz goes further than Kass in rejecting rationalism, not only refusing rationality’s final say over moral matters, but refusing it a seat at the table in the first place. Where Kass simply was a non-rationalist, Prinz is a proper a-rationalist about morality. For Prinz, a moral judgment’s being justified is equivalent to its resulting from an agent’s moral emotions. Any instance of disgust about human cloning or human manipulation of animal species is therefore sufficient to ground a moral belief about it, and many contradicting but valid moral beliefs can exist side by side in different agents. While one can always try and sway the emotional responses of others to more neatly align with one’s own, and thereby try to approximate a moral consensus, there are no means to actually *criticize* a person’s moral judgment and no standard to judge the appropriateness of moral emotions beyond comparing them to the rest of one’s own emotions and sentiments for compatibility (Prinz 2009, 120–25).

There are reasons to believe that this latter proposal is the theoretically sounder one compared to Kass’ approach, as it does not rely on emotions having genuine epistemic access to otherwise inaccessible facts. However, whichever horn of the dilemma one embraces, be it Kass’ incommunicable moral truth or Prinz’ equal validity of emotional moral judgments, something important is lost. On the one end, one loses the ability to communicate moral justification to people who have “different intuitive [affective] moral responses to the same issue” (Devolder et al. 2021, 436), and on the other end, one loses the notion of a fixed moral matter of fact altogether, making the whole enterprise of moral recommendation and admonishment pointless. For the bioethicist interested in improving the welfare of animals, both these options are bound to be unsatisfying; in either case, the bioethicist is, in effect, reduced to try and make emotional appeals.

Should this be the price for giving up rationalism in one’s evaluation of reactions of repulsion to cases such as that of the Belgian Blue? Our answer to this question is: It depends.

In the definition of rationalism, we have given above, we have split rationalism into two theses; one, PT, about human psychology, and our possessing this capacity of rationality, which is wholly divorced from emotions, and a second thesis, MT, about how that capacity relates to justifications in morality. The way we have phrased those theses has made it look seemingly obvious that the truth of the latter would partly depend on the truth of the former; only if the capacity of rationality as rationalists describe it actually exists can it play the justificatory role which they claim it plays. If the former is called into question—as we have claimed it is, both by empirical results and by philosophical reflection—then the latter must be as well. The rejection of this, second, thesis is what gets us into trouble in the application of the yuck factor. For, if moral justification does not happen in reference to something about our shared world that is objective, or at least not wholly subjective like the feeling of revulsion, then we are confined in our moral recommendations to those who share our subjective experience.

If one wants to avoid this isolating consequence while also taking seriously the mounting criticisms of rationalism’s conception of rationality, one would somehow have to be able to deny PT while affirming (at least a version of) MT. Against appearances, we think that this is possible. To show how, we will next look at a, in our view successful, application of the yuck factor by Midgley. Then, in the final section, we will give an account of what we think is responsible for the success of her application of the yuck factor, and how to replicate it.

## Mary Midgley on Emotions and the Yuck Factor

Before we show how Midgley uses the yuck factor in a way that takes seriously the problems with rationalism without dropping the kind of intersubjectively communicable morality implied by it, it will be helpful to say some things about how Midgley conceives of those problems, and of that morality.

Midgley is a non-rationalist in our sense, in that she denies at least one of the two central theses of rationalism. She denies PT, the thesis that there is a capacity called rationality which is independent from emotions and which is uniquely able to give us an accurate picture of the objective structure of the world uncoloured by our subjective phenomenology. This, she calls the “colonial picture” (1978, 260) of rationality, wherein reason is treated as a governor imposing order over the foreign lands of emotion. Rather than viewing rationality as a “single faculty in man” (Midgley 1983, 45) separable from emotions, she considers the distinction between the two to be a purely heuristic one which does not bear out in reality. In reality, Midgley holds, emotion and rationality form a continuity. To Midgley, rationality does not impose on emotions an order external to them; rather, emotions are rational just then when they are well-organized *as* emotions. Rationality, understood psychologically “is the process of *choosing which*” (1978, 258, emphasis in the original) in a conflict of emotions or desires. Rationality, in a sense, supervenes on emotion, as that kind of emotional constitution which coheres well with reality. Accordingly, rationality is neither a single faculty, nor is it wholly detached from emotions, nor does it give us unique access to the objective structure of the world. At least not in a way that is not always coloured by our subjective affective and emotional impressions. She therefore wholeheartedly rejects PT.

At the same time, Midgley does not categorically reject the historical rationalists as straightforwardly mistaken—though she holds the strict distinction between rationality and emotion to be merely heuristic, she takes it to have had merits as far as it went, and to have been broadly correct about a number of things. Among those is the rejection of subjectivism and relativism (Midgley 1993) and the conviction that “some preferences [are] ‘more rational’ than others” (Midgley 1978, 259). In other words, rationalism did right in supporting the deeply held intuitions of universality and necessity Birondo takes to be the original impetus for adopting rationalism in the first place.

Still, Midgley’s defence of the yuck factor is explicitly a defence against the overreach of a rationalism which tries to deny disgust a productive role in moral inquiry (Midgley 2003, 105). However, in contrast to Kass, her claim is not that, in addition to rational insights about morality, we should also take seriously a second, emotional, kind of insights, which are outside of rationality’s reach. Rather, her argument is that disgust and appeals to it *are not themselves irrational*, nor are they a-rational. Instead, they represent “real objections that can be spelled out, made clearer, and set against other considerations” (2003, 106). When we say that something is repugnant, or that it is monstrous or unnatural, these statements do not make an appeal to some blind instinct that can be dismissed as ‘mere’ emotion; nor do they express an attitude which alone is enough to pronounce them right in calling that thing morally bad. Rather, they do something more complicated: By calling something ‘disgusting’ or ‘unnatural,’ we are employing notions which have been charged, personally and culturally, with meaning which is sometimes too complex for us to consciously express to ourselves in full in the moment. Midgley takes such statements as the conscious, high-fidelity expressions of lower-fidelity thought which in further analysis can be revealed in more detail. In the case of gene-editing, and, likely, also of excessive breeding, what people are doing when they react with outrage is, according to Midgley, that “they are objecting to attacks on the concept of species” (2003, 105). That concept of species, in turn, is one which has been charged with information and value, such as the idea that when something rests within the boundaries of a species, it tends to be able to thrive autonomously in a favorable environment. For the Belgian Blue, our repugnance may point us precisely to the breakdown of this value we associate with natural species. We cannot immediately, and on first viewing, explain: ‘This animal has been changed in a way that promises disastrous consequences if they do not have access to human intervention.’ But by expressing disgust with it, we may be already gesturing to just such a suspicion.

If the claim behind such an expression is not fully rationalizable, that is not because it points us to the hidden wisdom of Kass’ heart, but because we are finite and fallible beings. Our cognitive apparatus does not always consciously and explicitly represent to us all the consequences it may unconsciously perceive implied by a situation, based both on the immediate data of the situation and on the complex cultural baggage of the notions involved. We often have to rely on non-conscious and, in a narrow sense, pre-reflective reactions to get our best picture of the world. To say, then, ‘this is disgusting’ or ‘this goes against nature’ when you see the Belgian Blue are substantive moral claims about which “others can understand what objection they are making even if they disagree” (Midgley 2003, 106), and which are therefore capable of being examined. Such examination can happen both by reflecting on the conditions under which such an affective judgment has been produced, such as the cultural background of the notion involved in it or by looking at the judging person’s personal history, but also by examining the kind of claims which are entailed by the judgment; whether it is the case that “there is a rational, conceptual link between [repugnant actions] and their [detrimental] results” (Midgley 2003, 104), so whether, in principle, one accepts negative consequences in acting in the repugnant way.

In whichever way one chooses to evaluate reactions of repugnance, two things are clear for Midgley: first, that disgust is capable of being evaluated, both because it makes an understandable point, and because that point is, in principle, accessible to rational thought. Second, in making that evaluation, one will always make use of the cognitive apparatus within which rationality can only exist in reference to desires and emotions which are more or less in line with reality. One will never be able to step ‘outside’ the affective perspective and look at an emotion with completely dispassionate rationality. This is because rationality is not merely informed by emotions, but essentially bound up with them, as “complementary aspects of a single process” (Midgley 2003, 105). Since being rational means nothing but having a well-ordered emotional life, the notion of purely unemotional rationality is nothing but a helpful fiction to distinguish calm from passionate reasoning.

How, then, does Midgley escape the problem of the lack of intersubjective communicability which Kass suffers from? Recall that we have criticized Kass for closing off proper moral justification to the people who happen to not have the right kind of constitution which would lead them to have the right kind of emotions. Kass, we have said, thereby fails to deliver the intersubjective communicability which we expect from morality. It seems that it is a valid criticism also of Midgley, that to anyone who does not have the correct emotions, crucial moral insights are simply inaccessible, making proper moral judgments just as impossible to reliably intersubjectively communicate as it was for Kass. However, with Midgley, having the correct kind of emotional constitution is both a much more permissive limit than it is for Kass and one that is much more in line with the limits we would expect to be placed on competent moral inquirers anyway. It is more permissive because in order to have the right emotional profile necessary for making proper moral judgments, for Midgley, one does not need to have a specific kind of character or have enjoyed a specific kind of education. Midgley does not exclude those who feel no immediate disgust in the face of biological engineering as unable to ever see why they are wrong—although she does hold that they *are* wrong. All one requires to competently make or evaluate judgments such as ‘The existence of the Belgian Blue is grotesque—it ought not be bred!’ is to be “within the range of emotional normality” (Midgley 1978, 272). She requires only a functional human emotional apparatus which is free from serious deficiencies such as the incapability of psychopaths for proper affective empathy (1978, 271).

So, Midgley’s requirements for someone competent in making proper moral judgments is more permissive than those implied by Kass’ position, where one is either revolted by the thought of human cloning, or one is unable to properly evaluate it at all. Her requirements are also more in line with what we would expect requirements for successful moral inquiry to be; for, if one falls out of this range of emotional normality, one does not only, as with Kass’ poorly conditioned inquirer, lack the ability to grasp certain pieces of moral wisdom, but one falls out of rationality more generally. If rationality is the “process of *choosing which*” (Midgley 1978, 258) between competing desires and emotions, then the failure to have a broadly normal range of desires and emotions entails an inability to be rational at all. This, presumably, will be accompanied by all the moral particularities associated with non-rational beings—psychopaths would be less blameworthy for example, and it would be more acceptable to restrict their freedoms for their own good and for the protection of others. So, Midgley ends up no more exclusionary than are rationalists. Like them, she considers any rational person to be a competent moral agent capable of accessing, in principle, any grounds for moral justification. She contends, further, that this requirement of “emotional normality” is not an unusually demanding one either. Rather, the assumption of “a common mental structure with other people” is “a necessary condition of our practical condition” (Midgley 1993, 80). She goes on to say:

We do not have to assume that [others] are in any way just like us, but we do have to assume, if we are to communicate with them at all, that there is an adequate likeness in basic structure. This is part of our general assumption of inhabiting a single world which is in principle coherent and intelligible—an assumption that is needed as much for science as for morals, and is indeed the basis of all thought. (ibid.)

Here, we can again see Midgley’s commitment to objectivity (albeit a relatively weak version of it). There is a common mental structure to all agents, which enables us to access the structure of the common world in which we live, mediated by our affective responses to it. This, rightly, may evoke what we have called above rationalism’s thesis about the nature of morality, MT, that the capacity which best gives us access to the objective structure of the world is what we ought to use to arrive at moral justification. This is, in essence, the lesson we should take from Midgley: that just because we dispute the characterization of rationality which rationalists base their project on, that does not mean that we have to abandon that project wholesale. We can, as it were, still work with a tempered version of rationalism.

Midgley, then, succeeds in applying the yuck factor where Kass and arguably Prinz fail: She manages to accommodate the manifest salience of reactions of disgust in moral justificatory discourses without abandoning the upshots associated with rationalism.

What does that mean for bioethicists interested in considering the moral implications of disgust-responses? This will be the subject of the last section of this article, where we will formalize what we think is productive in Midgley’s approach, and how bioethicists can use it.

## Why Midgley Succeeds where Kass Fails

The main reason that Midgley does not experience the same problems which Kass and Prinz experience in their application of the yuck factor is that she does not deny rationalism wholesale. Instead, she criticizes classical rationalism for operating on an outdated anthropology. The view of a human mind bifurcated into purely subjective, emotional faculties on the one hand which amount to “a kind of wholly contingent slop or flow” (Midgley 1978, 256), and a dispassionate, purely rational one on the other hand, she thought experience did not bear out. As we have discussed above, psychology and neuroscience have since then given added credence to her view. The psychological thesis of rationalism, PT, which assumes just such a capacity of pure rationality, she therefore rejects.

While she does temper rationalism in questioning its anthropological assumptions, Midgley does not, however, reject rationalism as a project. Like Birondo, Midgley understands rationalism to be a response to a set of expectations we have of the reality of moral thought. That expectation is that, to speak with Midgley, ‘we can make moral judgments’; we can defend our moral convictions against people who disagree with them and we feel justified in the hope that we can reliably communicate which one among competing moral judgments is, at least *prima facie*, the right one. Rationalism takes rationality, and its role in moral justification, to be a guarantor for the non-arbitrary and in-principle-communicable nature of which moral judgments are correct, and which are not. This is, in essence, what is expressed by what we have called above rationalism’s thesis about the nature of morality, MT, and this is the part of classical rationalism which Midgley’s tempered rationalism still retains. For, while Midgley sees the picture of rationality as a discrete capacity as untenable, the same is not true for rationality as a logical category: the ‘rationality’ of ‘a rational explanation’ or ‘a rationally ordered system’, that is, the idea of a logically consistent, and in-principle intelligible order of things, still remains. There is an intelligible, rational structure to our shared world, and our moral judgments must be justified with reference to that structure. But against the optimism of classical rationalists, to access it, it is not enough to be dispassionate in our examination. Things probably are not that simple and our epistemic access to the structure of our shared world will always be muddled and preliminary. But that does not mean that in morality, we are not after just such access, and that we are not justified in using our entire cognitive apparatus in constructing values and moral rules best in line with that reality. As part of that cognitive apparatus, disgust can be a helpful tool. It is a tool, however, which itself has to be subject to examination by that cognitive apparatus. We should not dismiss objections from repugnance as ‘merely’ emotional. Rather, we should examine them for their origin and for the substantive objections implied by them:

we need to supplement [feelings of repugnance] by thought, analysing their meaning and articulating them in a way that gives us coherent and usable standards. Unanalysed feelings sometimes turn out to be misplaced. Disgust can spring from chance associations or unfamiliarity or mere physical revulsion, such as a horror of cats. We always have to look below the surface. We must spell out the message of our emotions and see what they are trying to tell us. (Midgley 2003, 106)

So, neither should we restrict our moral justification to feelings of disgust, nor should we ignore those feelings. Instead, we should take such feelings seriously as the results of our cognitive apparatus, not all of the inner workings of which are always transparent to us. As such, they are revisable and subject to reflection by that same cognitive apparatus, again understood as a whole rather than cut up in an emotional part producing data and a rational one sorting it out.

For the person horrified by cats, she may find upon investigation that the origin of her feeling is a traumatic event in her childhood, or perhaps cannot be ascertained at all, which should make her suspicious of its salience. Even though she may remain disgusted by the cat, she may judge that disgust to be irrational in that it has no place in an accurate picture of reality as such and therefore also not in moral justification. On the other hand, and to come back to the example guiding this article, we can ask why we are disgusted by the thought of the Belgian Blue. The answer to that question may come out closer to Midgley’s “attacks on the concept of species” (2003, 105) and the predictably negative as well as the altogether unpredictable consequences of them on the health of the animals in question. Note that such an investigation will almost invariably rely also on the feelings at issue—only if we are sensitive to our disgust responses in recollection, for example, will we be able to locate the traumatic cat-event in our memory.

Crucially, this is no different than what we ought to do with calmer, more conscious hypotheses about the world: Even if we have come by an opinion only through conscious deliberation, we still are well-advised to analyse that process and its sources for potential biases or logical leaps, and in doing so, we should make use not only of dispassionate, calm reasoning, but our cognitive apparatus more broadly. Even if a judgment seems to come to us as a purely ‘rational’ intuition, we should not take that fact alone as enough to fully justify it—if that judgment, say, is accompanied by an intense feeling of revulsion, then we are well advised make use of that feeling to examine and, if applicable, revise the judgment.

Usually, for judgments based initially on disgust, such examination will not be as easy as reducing an affective response to some obvious improper association or clear harm. That is precisely the point: the world is messy and too complicated to be constantly well-represented by the most conscious parts of our cognition which roughly correspond to what rationalists would want to label as rationality. This means that we need to carefully consider the input of more affective, ‘hotter’ cognition on our judgments. We simply cannot afford to ignore it.

## Footnotes

1 We will be using the terms ‘disgust’ and ‘repugnance’ interchangeably throughout the text.

2 With the use of the phrase ‘in the world’, we do not mean to imply that rationalists are all moral realists. Singer’s mentor Richard M. Hare, for example, is both a rationalist and an anti-realist.

3 Another very fruitful attempt at this that bears mentioning here is Railton (2014).
